# Supplementary material for: Quinone Pool, a Key Target of Plant Flavonoids Inhibiting Gram-Positive Bacteria
Source: Molecules. 2023 Jun 24;28(13):4972. doi: 10.3390/molecules28134972 (PMC10343193; doi:10.3390/molecules28134972)
Supplement: Supplementary file 1 [file molecules-28-04972-s001.zip › molecules-2431057-supplementary.pdf]

Supplementary Materials

Figure S1. The HPLC-UV profiles of representative MK extract from *S. aureus* ATCC 25923.

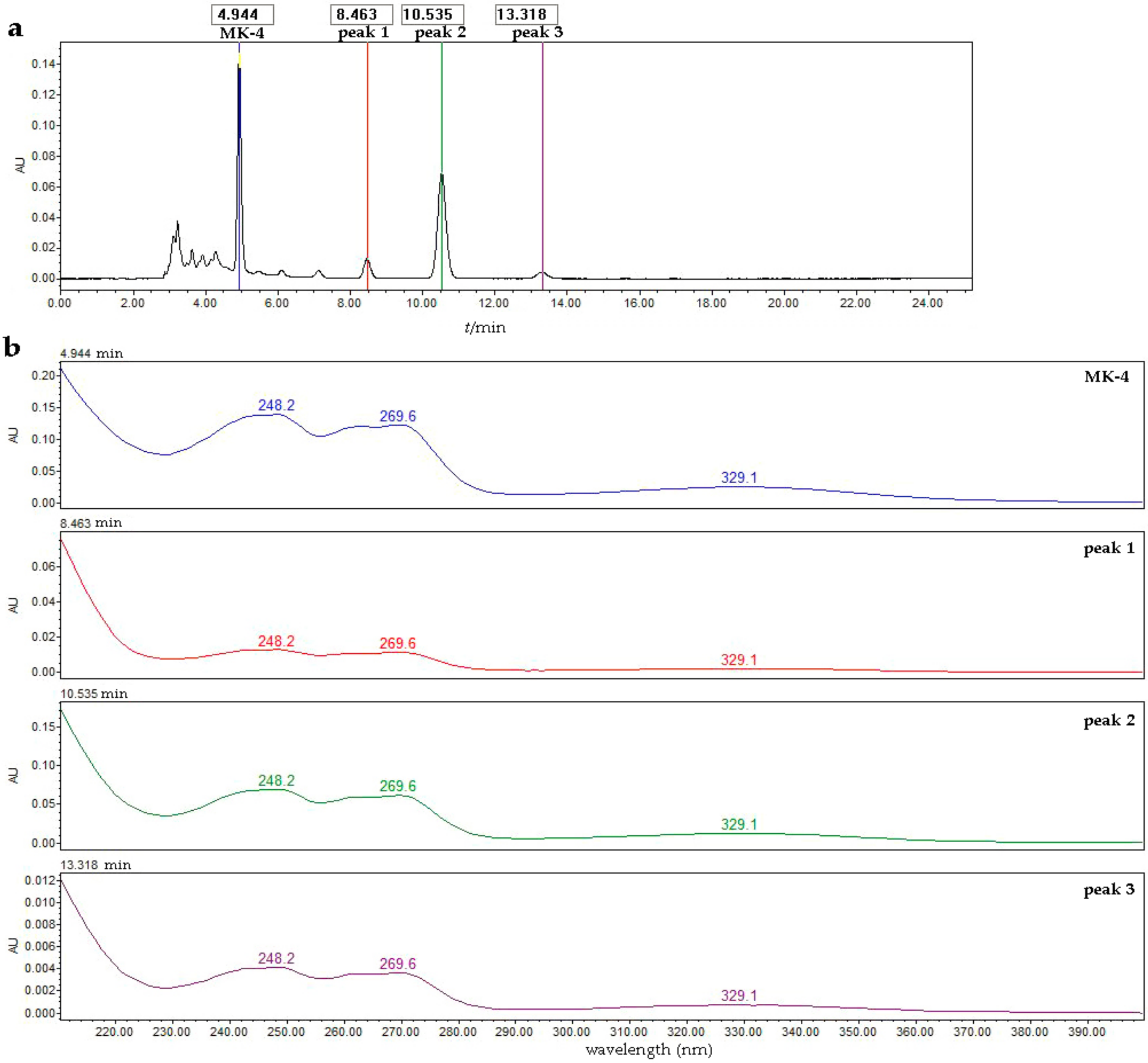

Figure S1. The HPLC-UV profiles of representative MK extract from *S. aureus* ATCC 25923. **a**, the HPLC of profile of MK extarct; **b**, the UV spectroscopies of four peaks respectively at 4.944 (MK-4 as an internal standard), 8.363 (peak 1), 10.535 (peak 2) and 13.318 (peak 3).
